# Preclinical insights into fucoidan as a nutraceutical compound against perfluorooctanoic acid-associated obesity *via* targeting endoplasmic reticulum stress

**DOI:** 10.3389/fnut.2022.950130

**Published:** 2022-08-12

**Authors:** Jiaqi Liu, Chao Guo, Yuqin Wang, Min Su, Wenjun Huang, Keng Po Lai

**Affiliations:** ^1^Key Laboratory of Environmental Pollution and Integrative Omics, Guilin Medical University, Education Department of Guangxi Zhuang Autonomous Region, Guilin, China; ^2^Department of Clinical Pharmacy, Guigang City People’s Hospital, The Eighth Affiliated Hospital of Guangxi Medical University, Guigang, China

**Keywords:** polysaccharides, mechanisms, obesity, immunomodulatory action, bioinformatics

## Abstract

Obesity is a growing global health problem; it has been forecasted that over half of the global population will be obese by 2030. Obesity is complicated with many diseases, such as diabetes and cardiovascular diseases, leading to an economic impact on society. Other than diet, exposure to environmental pollutants is considered a risk factor for obesity. Exposure to perfluorooctanoic acid (PFOA) was found to impair hepatic lipid metabolism, resulting in obesity. In this study, we applied network pharmacology and systematic bioinformatics analysis, such as gene ontology (GO) and Kyoto Encyclopedia of Genes and Genomes (KEGG) enrichment analyses, together with molecular docking, to investigate the targets of fucoidan for treating PFOA-associated obesity through the regulation of endoplasmic reticulum stress (ERS). Our results identified ten targets of fucoidan, such as glucosylceramidase beta (GBA), glutathione-disulfide reductase (GSR), melanocortin 4 receptor (MC4R), matrix metallopeptidase (MMP)2, MMP9, nuclear factor kappa B subunit 1 (NFKB1), RELA Proto-Oncogene, NF-KB Subunit (RELA), nuclear receptor subfamily 1 group I member 2 (NR1I2), proliferation-activated receptor delta (PPARD), and cellular retinoic acid binding protein 2 (CRABP2). GO and KEGG enrichment analyses highlighted their involvement in the pathogenesis of obesity, such as lipid and fat metabolisms. More importantly, the gene cluster is responsible for obesity-associated diseases and disorders, such as insulin resistance (IR), non-alcoholic fatty liver disease, and diabetic cardiomyopathy, *via* the control of signaling pathways. The findings of this report provide evidence that fucoidan is a potential nutraceutical product against PFOA-associated obesity through the regulation of ERS.

## Introduction

Obesity is a global health problem, especially in developed countries. According to the data from World Health Organization (WHO), the number of global obese people has tripled since 1975.^1^ More seriously, the prevalence of obesity among children and adolescents has risen dramatically from 4 to 18% in the last 40 years. It has been foreseen that 51% of the global population will be obese by 2030 (1). Obesity increases the risk of many dysfunctional metabolism disorders, such as diabetes mellitus, adiposis hepatica, dementia, and cancer (2), leading to imposing economic impacts on our society, such as medical costs and premature mortality costs (3, 4). The pathogenesis of obesity is multifactorial, such as genetic and environmental factors, changes in substance metabolism and endocrine function, fatty hypertrophy, abnormal neuropsychiatry, living, and eating habits (5). Cumulating epidemiological and animal studies suggested the contribution of environmental pollutants as a risk factor for obesity (6, 7). It is widely reported that exposure to environmental pollutants, such as bisphenols, polychlorinated biphenyls, and perfluorocarbons, is positively correlated to the development of obesity (6). Perfluorooctanoic acid (PFOA), a family member of perfluorocarbons, has been evidenced in an increase in body mass index (BMI) and obesity risk (8). A European Youth Heart Study indicates that childhood exposure to PFOA may induce adiposity and impair glucose metabolism (9). PFOA-induced mitochondrial dysfunction may be the main cause of obesity as a result of intercellular mitochondria-dependent function loss, which is closely related to fat deposition (10, 11). In addition, endoplasmic reticulum stress (ERS) exerts pathophysiological actions in the induction of insulin resistance (IR) and the development of obesity (12). ERS mediates lipotoxicity, dyslipidemia, and IR, indicating that ERS may be a potential pharmacological target against obesity (13). Although existing therapeutic option using slimming drugs is commercially available, the medication treating obesity has proven to be mostly resistant to treatment, marked by insufficient efficacy and uncertain safety (14). Thus, the discovery of nutraceutical compounds using natural ingredients could be an alternative way of treating obesity. Fucoidan, known as fucoid polysaccharides extracted from brown alga, was found to possess nutritive value and pharmacological activities, such as anticoagulant, antitumor, antithrombotic, antiviral, and antioxidant (15). *In vitro* study showed that fucoidan contributes to the reduction of lipid accumulation and the regulation of glucose consumption (16). It has been reported that fucoidan has effective anti-obesity effects *via* modulating IR, oxidative stress (OS), and gut microbiota *in vivo* (17, 18). However, pharmacological mechanisms involved in fucoidan against obesity are still largely unknown. In the present report, we applied network pharmacology and systematic bioinformatics analysis to reveal the beneficial effects and pharmacological mechanisms of fucoidan against PFOA-associated obesity through the regulation of ERS.

## Materials and methods

### Identification of fucoidan targets against perfluorooctanoic acid-associated obesity through the regulation of endoplasmic reticulum stress

The targets of fucoidan were identified by searching the databases, such as the Comparative Toxicogenomics Database (19), the SwissTargetPrediction database (20), the SuperPred database, and the PharmMapper database (21). The identified targets were subjected to the UniprotKB database (Swiss-Prot) for conversion to human genes (22). The ERS-related genes and PFOS-associated obese genes were obtained by searching the databases, such as the Online Mendelian Inheritance in Man (OMIM) database (23), the GeneCards database (24), and the National Center for Biotechnology Information (NCBI) database (25). The fucoidan target genes, the ERS-related genes, and the PFOS-associated obese genes were overlapped to determine the fucoidan targets against PFOA-associated obesity through the regulation of ERS. The STRING (version 11.0) tool was used to determine protein-protein interaction (PPI) of fucoidan targets against PFOA-associated obesity through the regulation of ERS (26). The Network Analyzer of Cytoscape_v3.8.2 was set under a median or maximum degree of freedom, the core targets were obtained under the upper limit of the screening range with a maximum degree value in topology data, and the lower limit was two times the median degree of freedom (27).

### Gene ontology and Kyoto Encyclopedia of Genes and Genomes enrichment analyses of laminarin targets against middle cerebral artery occlusion

The overlapped fucoidan target genes, the ERS-related genes, and the PFOS-associated obese genes were subjected to ClusterProfiler and GOplot packages in R-language software for the gene ontology (GO) and Kyoto Encyclopedia of Genes and Genomes (KEGG) enrichment analyses to determine the functional roles and mechanisms underlying fucoidan against PFOA-associated obesity through the regulation of ERS. The network of biological processes and the signaling pathways involved in fucoidan against PFOA-associated obesity was constructed by using the Cytoscape v3.8.2 tool (27).

### Molecular docking assay

Molecular docking analysis was used to assess the binding of fucoidan to its target proteins. Cytoscape was set under a median degree of freedom of 2 and a maximum degree of freedom of 3, the core protein targets were obtained at the range of median and maximum degrees of freedom. The protein structure of the core targets was obtained from the Protein Data Bank (PDB) database (28). The chemical structure of fucoidan was obtained from the PubChem database (29). The protein structure of matrix metallopeptidase (MMP)9, RELA Proto-Oncogene, NF-KB Subunit (RELA), and proliferation-activated receptor delta (PPARD) was obtained from the PDB database. Chem Bio Office 2010 software and Autodock Tools 1.5.6 were used to perform the molecular docking analysis (30). The PDB file was converted to a pdbqt file that can be recognized by the Autodock program. The docking parameter setting was assessed according to the root mean square deviation (RMSD) of the ligand molecule. In addition, the RMSD ≤ 4 Å was the threshold for the conformation of the ligand molecule. The results were displayed using PyMOL (version 2.3).

## Results and discussion

### Identification and functional characterization of fucoidan targets against perfluorooctanoic acid-associated obesity

**FIGURE 1:**
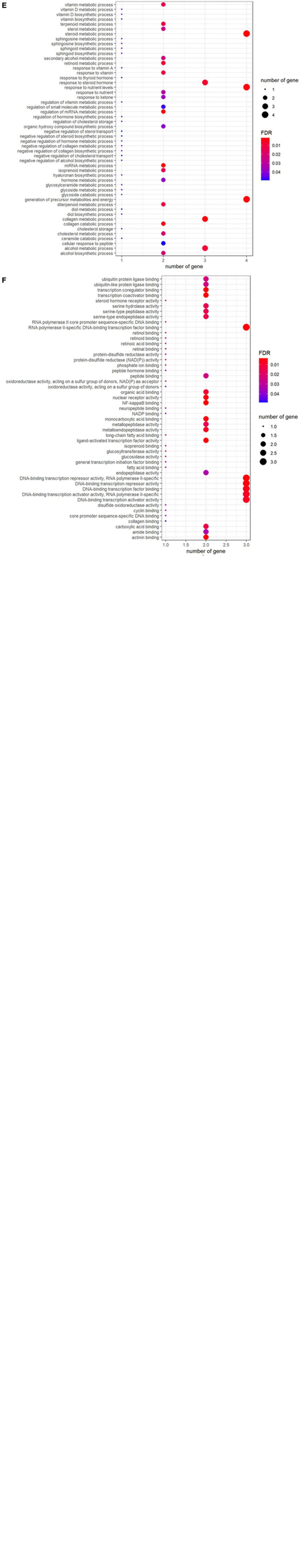
Identification and functional characterization of fucoidan’s targets against perfluorooctanoic acid (PFOA)-associated obesity through the regulation of endoplasmic reticulum stress. **(A)** A Venn diagram showed the number of shared fucoidan-, PFOA-induced obesity-, and endoplasmic reticulum stress-associated genes (left panel). The Protein–protein interaction of the ten core targets was analyzed by using STRING (right panel). Gene ontology enrichment analysis showed the involvement of fucoidan’s targets in biological processes related to **(B)** lipid and fat metabolisms, **(C)** oxidative stress and inflammatory responses, **(D)** cell proliferation and differentiation, **(E)** metabolisms and biosynthesis, and **(F)** molecular functions related to fat synthesis. The size of the bubble represented the number of involved genes and the color of the bubble represented the significance of the terms.

By searching the databases, a total of 363 fucoidan-associated genes, 258 PFOA-associated obese genes, and 4,293 ERS-related genes were identified (Figure 1A). When we overlapped these genes, we found 10 potential targets of fucoidan against PFOA-associated obesity through the regulation of ERS, such as glucosylceramidase beta (GBA), glutathione-disulfide reductase (GSR), melanocortin 4 receptor (MC4R), MMP2, MMP9, nuclear factor kappa B subunit 1 (NFKB1), nuclear receptor subfamily 1 group I member 2 (NR1I2), PPARD, RELA, and cellular retinoic acid binding protein 2 (CRABP2) (Figure 1A and Table 1). These targets were subjected to GO enrichment analysis to determine the functional roles of fucoidan against PFOA-associated obesity. Our results highlighted the biological processes related to biosynthesis and metabolisms of lipid and fat, such as regulation of lipid metabolic process, fatty acid oxidation, cellular lipid catabolic process, lipid modification, lipid glycosylation, cellular response to lipopolysaccharide, and regulation of lipid storage (Figure 1B). In addition, we found fucoidan’s targets in response to OS and hepatocyte growth factor and its involvement in inflammatory responses, such as the response to interleukin (IL)-6 and IL-1, the regulation of IL-12 production, the regulation of cytokine production, and leukocyte migration (Figure 1C). In addition, the fucoidan’s target was found to contribute to cell proliferation and differentiation (Figure 1D) and biosynthesis and metabolisms (Figure 1E). In the molecular function analysis, we observed the highlights of glucose- and lipid-related functions, such as glucosidase activity, glucosyltransferase activity, long-chain fatty acid binding, and fatty acid binding (Figure 1F). Taken together, our data suggested that fucoidan could target the genes that are closely associated with the development of obesity.

**TABLE 1:** Core targets of fucoidan against perfluorooctanoic acid (PFOA)-associated obesity *via* targeting endoplasmic reticulum stress.

Gene name	Gene symbol	ENTREZ ID
Glucosylceramidase Beta	GBA	2629
Glutathione-Disulfide Reductase	GSR	2936
Melanocortin 4 Receptor	MC4R	4160
Matrix Metallopeptidase 2	MMP2	4313
Matrix Metallopeptidase 9	MMP9	4318
Nuclear Factor Kappa B Subunit 1	NFKB1	4790
Nuclear Receptor Subfamily 1 Group I Member 2	NR1I2	8856
Peroxisome Proliferator Activated Receptor Delta	PPARD	5467
RELA Proto-Oncogene, NF-KB Subunit	RELA	5970
Cellular Retinoic Acid Binding Protein 2	CRABP2	1382

### Fucoidan targeted the genes involved in signaling pathways of fat metabolisms and obesity-related disorders

**FIGURE 2:**
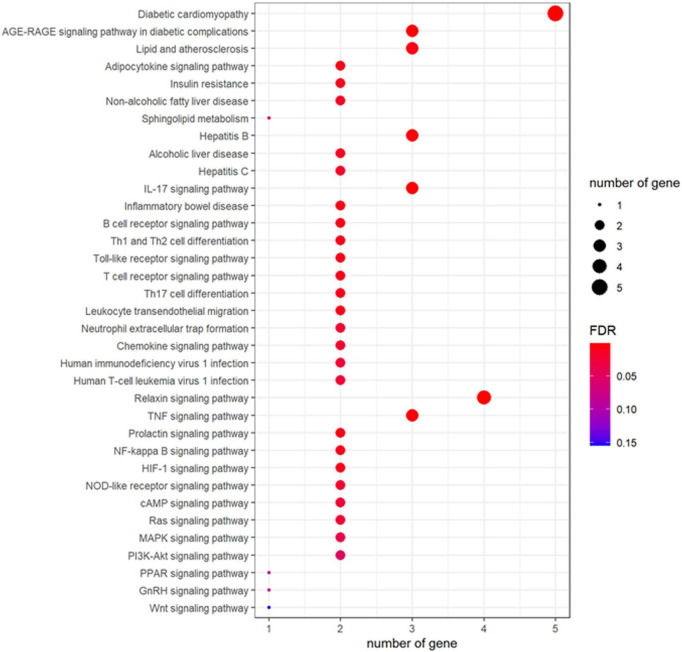
Fucoidan’s targets controlled the signaling pathways involved in the pathogenesis of obesity and its associated diseases. The size of the bubble represented the number of involved genes and the color of the bubble represented the significance of the terms.

To further determine the signaling pathways controlled by fucoidan’s targets, the KEGG pathway analysis was conducted. Our result showed that fucoidan could target the genes involved in fat metabolisms, such as the adipocytokine signaling pathway and the sphingolipid metabolism (Figure 2). In addition, many signaling pathways related to obesity, such as the Relaxin signaling pathway, the tumor necrosis factor (TNF) signaling pathway, the Prolactin signaling pathway, the NF-kappa B signaling pathway, the hypoxia-inducible factor-1 (HIF)-1 signaling pathway, the nucleotide-binding oligomerization domain (NOD)-like receptor signaling pathway, the cyclic adenosine 3’,5’-monophosphate (cAMP) signaling pathway, the Ras signaling pathway, the mitogen-activated protein kinase (MAPK) signaling pathway, the phosphatidylinositol-3-kinase (PI3K)-Akt signaling pathway, the proliferator-activated receptor (PPAR) signaling pathway, the gonadotropin-releasing hormone (GnRH) signaling pathway, and the Wnt signaling pathway, were highlighted in our result. The possible outcomes were obesity-related disorders, such as IR, non-alcoholic fatty liver disease, alcoholic liver disease, diabetic cardiomyopathy, and atherosclerosis (Figure 2). In addition, fucoidan’s targets were found to be involved in signaling pathways of immune responses, such as the IL-17 signaling pathway, the B-cell receptor signaling pathway, the Toll-like receptor signaling pathway, the T-cell receptor signaling pathway, the chemokine signaling pathway, and Th1 and Th2 cell differentiation (Figure 2). Collectively, our result highlighted the contribution of fucoidan’s targets in obesity-associated disorders.

### Direct binding of fucoidan to its target proteins MMP9, RELA, and proliferation-activated receptor delta

**FIGURE 3:**
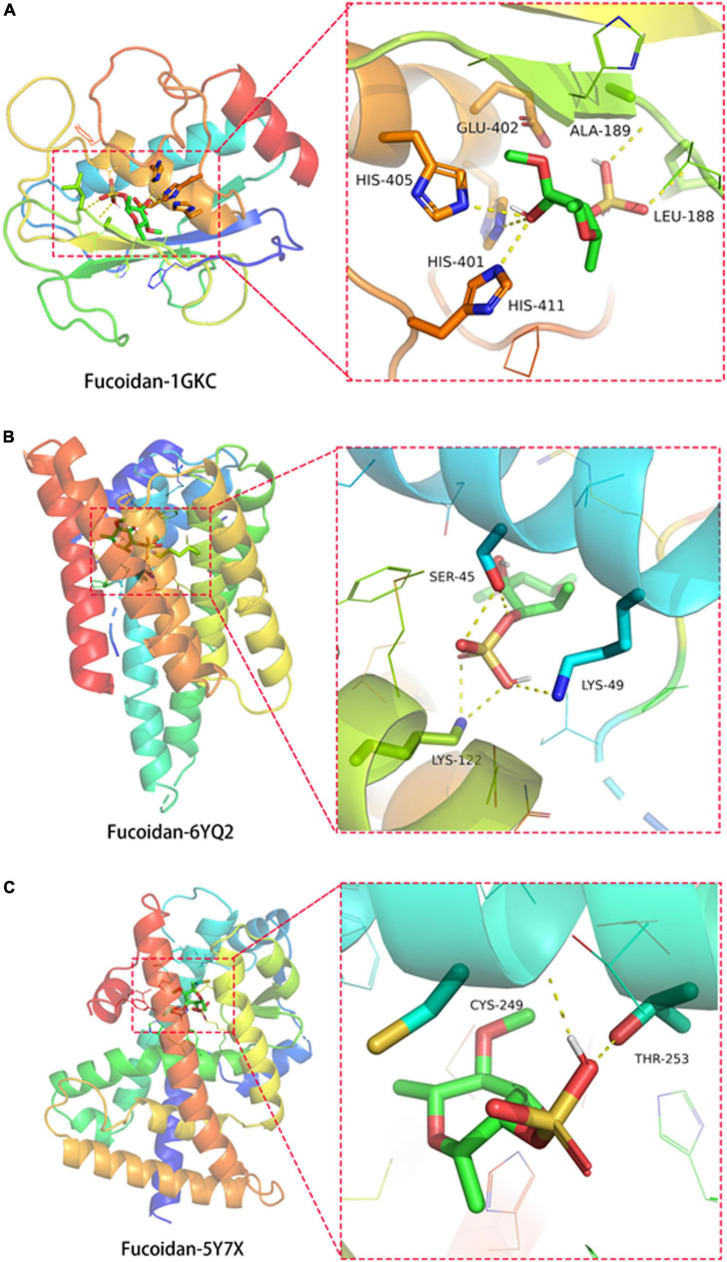
Direct binding of fucoidan to its target proteins matrix metallopeptidase 9 (MMP9), RELA Proto-Oncogene, NF-KB Subunit (RELA), and peroxisome proliferator-activated receptor delta (PPARD). Molecular docking showed the binding of fucoidan to **(A)** MMP9 (ID: 1GKC), **(B)** RELA (ID: 6YQ2), and **(C)** PPARD (ID: 5Y7X).

In order to investigate the possible direct binding of fucoidan to its target proteins, a molecular docking analysis was performed. First, the protein targets were prioritized using the Cytoscape v3.8.2 tool. Three top-ranked core proteins, such as MMP9, RELA, and PPARD, were identified. The protein structures of MMP9 (ID: 1GKC) (31), RELA (ID: 6YQ2) (32), and PPARD (ID: 5Y7X) (33) were obtained from the PDB database. Their binding affinities with fucoidan were determined using the AutoDock Vina program. The negative value of the binding affinity represented the possible direct binding of fucoidan to its target proteins. We found the formation of hydrogen bonds between fucoidan with amino acid residues of HIS-401 (2.8 Å), HIS-405 (3.1 Å), HIS-411 (2.8 Å), GLU-402 (2.6 Å), LEU-188 (2.9 Å), and ALA-189 (2.6 Å) of MMP9 (ID: 1GKC) (Figure 3A). The binding affinity was −6.1 Kcal/mol. For the RELA (ID: 6YQ2), its amino acid residues LYS-49 (3.3 Å), LYS-122 (3.0 Å), and SER-45 (2.8 Å) were found to form hydrogen bonds with fucoidan, and the binding affinity was −5.8 Kcal/mol (Figure 3B). Similar bindings were observed between fucoidan and PPARD (ID: 5Y7X) through the amino acid residues of CYS-249 (2.2 Å) and THR-253 (2.7 Å) with −6.7 Kcal/mol (Figure 3C). Taken together, our data suggested that fucoidan potentially binds to its target proteins directly.

## Discussion

In this report, we applied network pharmacology and bioinformatics analysis, such as GO and KEGG enrichment analyses, and molecular docking to investigate the possible use of fucoidan as a nutraceutical product for treating PFOA-associated obesity through the regulation of ERS. First, the result of network pharmacology identified 10 potential targets, such as GBA, GSR, MC4R, MMP2, MMP9, NFKB1, RELA, NR1I2, PPARD, and CRABP2, of fucoidan against PFOA-associated obesity. Network pharmacology is a common approach to drug discovery for metabolic disorders that include obesity (34, 35). Most of the identified targets were reported to be correlated with obesity and obesity-associated diseases.

Glutathione-disulfide reductase, also known as glutathione reductase (GR) (36), is considered a marker of antioxidant defense in patients with type 2 diabetes (37). Obesity is a prevalent cause of OS and ERS through the regulation of TCA cycle activity (38, 39). A cross-sectional study conducted in Serbia suggested that GSR is one of the antioxidant defense parameters in obese students with increased cardiovascular risk (40). MC4R is reported to play a significant role in energy balance and weight control (41), and inherited MC4R variant is one of the causes of obesity. A genetics associated study showed that MC4R gene variants are common in childhood obesity in the Turkish population (41). Another study by Martinelli’s group suggested that MC4R deficiency is correlated to increased fasting insulin levels and accelerated growth phenotypes (42).

Our result also demonstrated that matrix metalloproteinase family members, MMP-2 and MMP-9, were potential targets of fucoidan against PFOA-associated obesity. A correlation study in children with obesity hypertension showed the induction of plasma levels of MMP-2 and MMP-9 in patients with obese (43). More importantly, increased MMP2 and MMP9 were found to increase the risks of cardiovascular disease and clinical hypertension in children with obesity (44, 45). NFKB signaling is an important factor for inflammatory responses in obesity (46). As the key regulators of NFKB signaling, NFKB and RELA were believed as potential targets to overcome the problem of obesity. A gene interactions study demonstrated the involvement of the NFKB signaling pathway in obesity pathogenesis (47). It is further supported by Bauman-Fortin’s group that NFKB1 genotypes were associated with BMI and waist circumference (48). In addition, our result suggested that PPARD is a target of fucoidan against PFOA-associated obesity. Although most of the studies focused on PPARα activation in response to PFOA exposure (49), there are limited reports that demonstrated the association between PPARD and PFOA (50). Therefore, this gene cluster could be a promising target of fucoidan for treating PFOA-associated obesity.

In the later part of the study, the enrichment analysis of the fucoidan’s targets highlighted their importance in different biological processes. In the analysis, we focused on the functions and pathways related to the pathogenesis of obesity and its associated diseases. Our result highlighted many lipid-related metabolisms controlled by the fucoidan’s targets. The alteration of lipid metabolism is one of the causes of obesity, because obesity was associated with increased basal lipolysis in adipose tissue and increased circulating fatty acids (51). In addition, the altered lipid metabolism was reported to be linked with many signaling pathways, such as TNF signaling (52), Wnt signaling (53), and MAPK (54), leading to worse outcomes that included T2D and non-alcoholic fatty liver disease (55). All these signaling pathways were found to be targeted by fucoidan.

Finally, we conducted molecular docking to investigate the interaction of fucoidan with its targets. Molecular docking is a tool commonly used to predict the possible interactions between two molecules and is a powerful approach for structure-based drug discovery (56). Some studies used molecular docking for the drug discovery of fucoidan. For instance, Etimad’s group conducted molecular docking to unfold the anti-inflammatory potential of fucoidan for atherosclerosis (57). A similar approach was used to understand the anti-viral, antioxidative, and anti-diabetic roles of fucoidan (58–60). In addition, our result showed a high binding affinity of fucoidan to MMP9, RELA, and PPARD through the formation of hydrogen bonds, suggesting the drug-protein interaction and the anti-obesogenic ability of fucoidan (61). However, further validation is needed to confirm this prediction.

## Conclusion

Our results provide evidence that fucoidan is a promising nutraceutical compound for treating PFOA-associated obesity. The findings support the previous animal study that fucoidan prevents high-fat diet-induced obesity and inflammation by suppressing fat accumulation (62, 63). More importantly, our data delineate the molecular mechanism underlying the anti-obesogenic effect of fucoidan by targeting a cluster of genes involved in the pathogenesis of obesity and its related diseases. Furthermore, our results suggest the possible use of nutraceutical compound to improve the human health by reducing the toxicity of environmental pollutants. However, further preclinical study is needed to warrant the findings before the clinical use of fucoidan.

## Data availability statement

The original contributions presented in this study are included in the article/supplementary material, further inquiries can be directed to the corresponding authors.

## Author contributions

MS, WH, and KL conceived and designed the study and prepared the manuscript. JL and CG performed the data analysis and data interpretation. JL, CG, and YW conducted the bioinformatics and statistical analyses. All authors contributed to the article and approved the submitted version.

## Footnotes

1
https://www.who.int/news-room/fact-sheets/detail/obesity-and-overweight
